# Six Degrees of Freedom Displacement Measurement System for Wafer Stage Composed of Hall Sensors

**DOI:** 10.3390/s18072030

**Published:** 2018-06-25

**Authors:** Bo Zhao, Weijia Shi, Jiawei Zhang, Ming Zhang, Xue Qi, Jiaxin Li, Feng Li, Jiubin Tan

**Affiliations:** School of Electrical Engineering and Automation, Harbin Institute of Technology, D-403 Science Park, 2 Yikuang Street, Harbin 150080, China; hitzhaobo@hit.edu.cn (B.Z.); hit_zhangjiawei@163.com (J.Z.); 17S001042@stu.hit.edu.cn (M.Z.); 17S001051@stu.hit.edu.cn (X.Q.); 1140110416@hit.edu.cn (J.L.); 1140110428@hit.edu.cn (F.L.); jbtan@hit.edu.cn (J.T.)

**Keywords:** Hall sensor, displacement measurement, the least squares method, ellipsoid function

## Abstract

This paper proposes a decoupled six degrees of freedom (DOF) displacement measurement methodology, which is accomplished by utilizing six pairs of permanent magnets and six Hall sensors. Firstly, the coordinate transformation was mathematically derived, which represented the relationships between the main coordinate system of the motion system and each body coordinate system of the Hall sensors. With the aid of an ellipsoid function and the least squares method, only the output voltages of the six Hall sensors were required to decouple the six-DOF displacement and inclination of the motion platform with high accuracy. Finally, the experimental measurements demonstrate the effectiveness of the six DOF displacement measurement methodology, based on which the maximum errors of displacements can reach 0.23 mm and the maximum errors of inclinations can reach 0.07°.

## 1. Introduction

A multiple degree-of-freedom (Multi-DOF) measurement system is the key part in the precision stage, which is playing a critical role in many industrial applications in terms of integrated circuit (IC) manufacturing and optical components production. The increasing demands of industrial production such as lithography put stricter requirements on the precision stage, especially on the accuracy of its multi-DOF movement [1].

Sensors based on the Hall effect are widely used in different areas [2], including the position, the velocity, and the magnetic fields measurement. Hall-effect sensors can be used as position feedback devices [3]. The different position and direction of Hall effect sensors and permanent magnets generate different Hall effects [4]. A Hall sensor has an offset voltage, such that Petruk [5] presented a novel method of offset voltage minimization. Lozanova presented a novel three-axis sensor for simultaneous and independent magnetic-field component measurement based on the Hall effect [6], and subsequently constructed a novel single-chip sensing device [7]. The calibration of the multi-axes measurement by Hall sensor is another critical issue, because the cross-sensitivity among the measurement axes would limit the accuracy of the measurement system. To solve this problem, Wouters [8] presented a calibration scheme for a 3D Hall sensor. A three DOF and four DOF displacement measurement system has been proposed [9,10]. To measure translational displacement motions and angular motions of a six DOF planar motion stage, Li [11] presented a multi-axis surface encoder. Lee reviewed the current state-of-the-art techniques for multi-degree-of-freedom motion error measurement in a linear stage using a laser encoder-implemented system, and discussed the kinds of new science upcoming in the next few years [12]. Mura [13] proposed a new detection strategy suitable for Micro-Electro Mechanical System (MEMS) sensors with up to six degrees of freedom. A novel sensing methodology using two-axis Hall-effect sensors is proposed, which has the capability of micrometer-order positioning resolution, as well as an unrestricted translational and rotational range in planar 3-DOF motions, with potential capability of measuring all 6-DOF motions [14]. Sun [15] built the measurement system consisting of hall sensors and a cylindrical permanent magnet, and the experiment results demonstrate the system has satisfactory performance.

This paper is aimed at proposing a decoupled and precise six degree of freedom measurement method, which is accomplished by utilizing six pairs of permanent magnets and six Hall sensors for precision positioning. Firstly, the coordinate transformation is mathematically derived, which represents the relationships between the main coordinate system of the motion system and each body coordinate system of the Hall sensors. The novel measurement methodology is subsequently proposed to clarify the decoupling relationship between the output voltages of Hall sensors and the displacement of the chuck based on the least squares method. In addition, with the aid of the ellipsoid function, we fit the practical field of the permanent magnet at a certain plane height, which can be substituted for the output voltage of the Hall sensor. Finally, the experiment results demonstrate the effectiveness of the six DOFs displacement measurement methodology.

## 2. Description of the Measurement System

**Figure 1:**
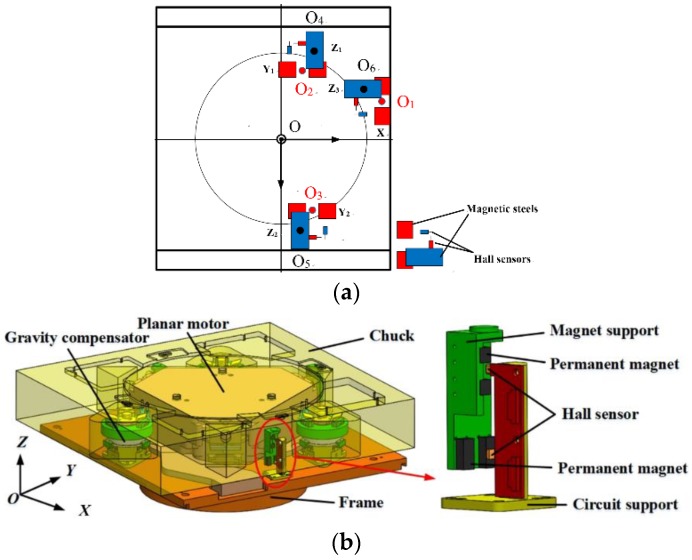
Schematic and structural diagram of short stroke (SS) stage with a six DOF Hall sensor displacement measurement system: (**a**) schematic diagram; (**b**) structural diagram.

The schematic and structural diagram of the short stroke (SS) stage is shown in Figure 1. In Figure 1a, red represents the horizontal direction and blue represents the vertical direction. As shown in Figure 1b, the SS stage is a magnetic suspension system. The chuck is suspended by three gravity compensators (GC) based on magnetic levitation. The vertical three DOFs, which are *Z*-axis displacement, the angle of rotation about the *X*-axis, and the angle of rotation about the *Y*-axis, can be activated by the three GCs. Each GC can provide adequate electromagnetic force along *Z-*axis for compensating the negative influence of the gravity on positioning. The horizontal three DOFs, which are *X*-axis and *Y*-axis displacements and the angle of rotation about the *Z*-axis, can be activated by the planar motor. The six DOFs of the chuck are measured by three groups of Hall sensors, each group of Hall sensors is composed of two pairs of permanent magnets which are bonded on the magnet support and two sensors mounted on the circuit support. The upper sensor can measure the vertical displacement of the chuck, and the lower sensor can measure the horizontal displacement. The travel range of the six DOF are 2 mm in *X* and *Y*, 800 μm in *Z*, 1 mrad in the angle of rotation about the *X*-axis, *Y*-axis, and *Z*-axis.

The installation of the Hall sensors and the distribution of the magnetic field on the magnet surface at different height have been given in [10]. The micro-coordinates coordinate system is *O-XYZ*, and the coordinate origin is at the center of mass of the micro-motion station. The coordinates of the symmetrical center point of each pair of permanent magnets in the micro-motion coordinate system are (*X_O_*_1_,*Y_O_*_1,_*Z_O_*_1_), (*X_O_*_2_,*Y_O_*_2,_*Z_O_*_2_), (*X_O_*_3_,*Y_O_*_3,_*Z_O_*_3_), (*X_O_*_4_,*Y_O_*_4,_*Z_O_*_4_), (*X_O_*_5_,*Y_O_*_5,_*Z_O_*_5_), (*X_O_*_6_,*Y_O_*_6,_*Z_O_*_6_); the sub-coordinate systems of each Hall sensor are established with the center points of each pair of permanent magnets as the origin: *O*_1_*-X*_1_*Y*_1_*Z*_1_, *O*_2_*-X*_2_*Y*_2_*Z*_2_, *O*_3_*-X*_3_*Y*_3_*Z*_3_, *O*_4_*-X*_4_*Y*_4_*Z*_4_, *O*_5_*-X*_5_*Y*_5_*Z*_5_, *O*_6_*-X*_6_*Y*_6_*Z*_6_.

Assume that the initial coordinates of each Hall sensor in each sub-coordinate system are (*x*_1*c*_,*y*_1*c*_,*z*_1*c*_), (*x*_2*c*_,*y*_2*c*_,*z*_2*c*_), (*x*_3*c*_,*y*_3*c*_,*z*_3*c*_), (*x*_4*c*_,*y*_4*c*_,*z*_4*c*_), (*x*_5*c*_,*y*_5*c*_,*z*_5*c*_), (*x*_6*c*_,*y*_6*c*_,*z*_6*c*_), which are (*X*_1*c*,_*Y*_1*c*_,*Z*_1*c*_), (*X*_2*c*_,*Y*_2*c*_,*Z*_2*c*_), (*X*_3*c*_,*Y*_3*c*_,*Z*_3*c*_), (*X*_4*c*_,*Y*_4*c*_,*Z*_4*c*_), (*X*_5*c*_,*Y*_5*c*_,*Z*_5*c*_), (*X*_6*c*_,*Y*_6*c*_,*Z*_6*c*_) in the micro-coordinate system. After the translation movement and rotation of the chuck, the coordinates of the Hall sensors in the sub-coordinate system turn into (*x*_1_,*y*_1_,*z*_1_), (*x*_2_,*y*_2_,*z*_2_), (*x*_3_,*y*_3_,*z*_3_), (*x*_4_,*y*_4_,*z*_4_), (*x*_5_,*y*_5_,*z*_5_), (*x*_6_,*y*_6_,*z*_6_), which turn into (*X*_1_,*Y*_1_,*Z*_1_), (*X*_2_,*Y*_2_,*Z*_2_), (*X*_3_,*Y*_3_,*Z*_3_), (*X*_4_,*Y*_4_,*Z*_4_), (*X*_5_,*Y*_5_,*Z*_5_), (*X*_6_,*Y*_6_,*Z*_6_) in the micro-coordinate system. The sub-coordinate system can be converted into the *O-XYZ* coordinate system by:

(1) $$\left[X_{i}\;Y_{i}\;Z_{i}\right]=\left[x_{i}\;y_{i}\;z_{i}\right]H_{i}\;+\left[X_{Oi}\;Y_{Oi}\;Z_{Oi}\right]\;i=1,2,\cdots ,6$$

$$\begin{array}{l} H_{1}=\left[\begin{array}{ccc} 0 & 1 & 0\\ 0 & 0 & -1\\ -1 & 0 & 0 \end{array}\right]\;H_{2}=\left[\begin{array}{ccc} 1 & 0 & 0\\ 0 & 0 & -1\\ 0 & 1 & 0 \end{array}\right]\;H_{3}=\left[\begin{array}{ccc} 1 & 0 & 0\\ 0 & 0 & 1\\ 0 & -1 & 0 \end{array}\right]\\ H_{4}=\left[\begin{array}{ccc} 0 & 0 & 1\\ -1 & 0 & 0\\ 0 & -1 & 0 \end{array}\right]\;H_{5}=\left[\begin{array}{ccc} 0 & 0 & 1\\ 0 & 1 & 0\\ -1 & 0 & 0 \end{array}\right]\;H_{6}=\left[\begin{array}{ccc} 0 & 0 & 1\\ 0 & -1 & 0\\ 1 & 0 & 0 \end{array}\right] \end{array}$$

Assume that the SS stage rotates around the *X*-axis at an angle *θ_x_*, rotates around the *Y*-axis at an angle *θ_y_*, and rotates around the *Z*-axis at an angle *θ_z_*, and then translates along the vector $\vec{M}=\left(X_{m},Y_{m},Z_{m}\right)$:

(2) $${\left[\begin{array}{c} X_{i}\\ Y_{i}\\ Z_{i} \end{array}\right]}^{T}={\left[\begin{array}{c} X_{ic}\\ Y_{ic}\\ Z_{ic} \end{array}\right]}^{T}\left[\begin{array}{ccc} 1 & 0 & 0\\ 0 & \cos\theta_{x} & \sin\theta_{x}\\ 0 & -\sin\theta_{x} & \cos\theta_{x} \end{array}\right]\left[\begin{array}{ccc} \cos\theta_{y} & 0 & -\sin\theta_{y}\\ 0 & 1 & 0\\ \sin\theta_{y} & 0 & \cos\theta_{y} \end{array}\right]\left[\begin{array}{ccc} \cos\theta_{z} & \sin\theta_{z} & 0\\ -\sin\theta_{z} & \cos\theta_{z} & 0\\ 0 & 0 & 1 \end{array}\right]+{\left[\begin{array}{c} X_{m}\\ Y_{m}\\ Z_{m} \end{array}\right]}^{T}\;i=1,2,\cdots ,6$$

Assume that *θ_x_*, *θ_y_,* and *θ_z_* are all small angles, then there are:

(3) $${\left[\begin{array}{c} X_{i}\\ Y_{i}\\ Z_{i} \end{array}\right]}^{T}={\left[\begin{array}{c} X_{ic}\\ Y_{ic}\\ Z_{ic} \end{array}\right]}^{T}\left[\begin{array}{ccc} 1 & \theta_{z} & -\theta_{y}\\ \theta_{x}\theta_{y}-\theta_{z} & \theta_{x}\theta_{y}\theta_{z}+1 & \theta_{x}\\ \theta_{x}\theta_{z}+\theta_{y} & \theta_{y}\theta_{z}-\theta_{x} & 1 \end{array}\right]+{\left[\begin{array}{c} X_{m}\\ Y_{m}\\ Z_{m} \end{array}\right]}^{T}\;i=1,2,\cdots ,6$$

The coordinates of the symmetrical center point of each pair of permanent magnets in the micro-motion coordinate system can be experimentally measured, as follows (unit: mm):

(4) $$\left\{ \begin{array}{l} \left(X_{O1},Y_{O1},Z_{O1}\right)=\left(197.35,53.35,-72.72\right)\\ \left(X_{O2},Y_{O2},Z_{O2}\right)=\left(-10.65,178.65,-72.72\right)\\ \left(X_{O3},Y_{O3},Z_{O3}\right)=\left(-17.85,-178.65,-72.72\right)\\ \left(X_{O4},Y_{O4},Z_{O4}\right)=\left(194.70,56.00,-37.95\right)\\ \left(X_{O5},Y_{O5},Z_{O5}\right)=\left(-8.00,181.30,-37.95\right)\\ \left(X_{O6},Y_{O6},Z_{O6}\right)=\left(-20.50,-181.30,-37.95\right) \end{array}\right.$$

and the coordinates of the initial points of each Hall sensor in the micro-motion coordinate system (unit: mm):

(5) $$\left\{ \begin{array}{l} \left(X_{1c},Y_{1c},Z_{1c}\right)=\left(191.35,53.35,-72.72\right)\\ \left(X_{2c},Y_{2c},Z_{2c}\right)=\left(-10.65,184.65,-72.72\right)\\ \left(X_{3c},Y_{3c},Z_{3c}\right)=\left(-17.85,-184.65,-72.72\right)\\ \left(X_{4c},Y_{4c},Z_{4c}\right)=\left(194.70,50.00,-37.95\right)\\ \left(X_{5c},Y_{5c},Z_{5c}\right)=\left(-14.00,181.30,-37.95\right)\\ \left(X_{6c},Y_{6c},Z_{6c}\right)=\left(-14.50,-181.30,-37.95\right) \end{array}\right.$$

Therefore, after the translational movement and rotation of the SS stage, the coordinates of the Hall sensors in the sub-coordinate system can be described as:

(6) $$\left[x_{i}\;y_{i}\;z_{i}\right]=\left(\left[X_{ic}\;Y_{ic}\;Z_{ic}\right]G+\left[X_{m}\;Y_{m}\;Z_{m}\right]-\left[X_{Oi}\;Y_{Oi}\;Z_{Oi}\right]\right)H_{i}^{-1}\;i=1,2,\cdots ,6$$

where

$$G=\left[\begin{array}{ccc} 1 & \theta_{z} & -\theta_{y}\\ \theta_{x}\theta_{y}-\theta_{z} & \theta_{x}\theta_{y}\theta_{z}+1 & \theta_{x}\\ \theta_{x}\theta_{z}+\theta_{y} & \theta_{y}\theta_{z}-\theta_{x} & 1 \end{array}\right]$$

## 3. Model Derivation

The output voltage of a Hall sensor depends on the magnetic field through it. The formula representing the relationship between the output voltage of the Hall sensor and the corresponding position in the magnetic field can be expressed by [10]:

(7) $$V_{Hall}=F\left(x,y,z\right)$$

Assume that the sensor rotates the angle *φ* about the *X*-axis, and the magnetic flux density perpendicular to the sensor plane is *B = B_z_ cosφ* − *B_y_ sinφ ≈ B_z_* − *B_y_ φ*, where |*φ*| ≤ 0.001. It can be subsequently considered that the magnetic induction perpendicular to the sensor plane is still *B_z_*. In a similar fashion, when the sensor rotates the angle *ψ* about the *Y*-axis, the magnetic induction perpendicular to the sensor plane can still be considered as *B_z_*. Therefore, the output voltage of the sensor is equal to the measured voltage when the sensor is rotated in a small angle around the horizontal plane and the sensor is placed horizontally in the same position.

**Figure 2:**
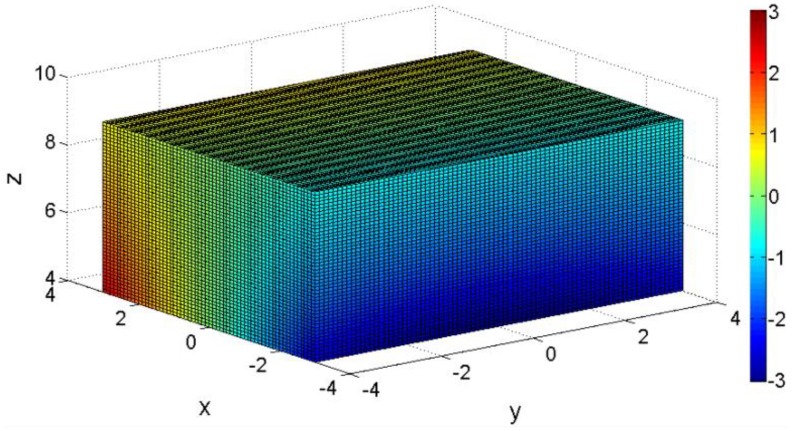
Distribution of voltage equivalent points in space plotted by Matlab R2014a.

Figure 2 indicates the measurements of the output voltage of the Hall sensors in the *O-XYZ* coordinate system. The strength of the magnetic field is represented by the absolute value of the voltage, and different colors indicate different output voltages. Three types of plane conic curves (elliptic, parabolic, and hyperbolic) have been given previously in Reference [10].

To analytically and accurately describe the relationship between the output voltages of the Hall sensors and the coordinates, an ellipsoid function is applied as follows:

(8) $$\frac{{\left(x-a\right)}^{2}}{b}+\frac{y^{2}}{c}+\frac{{\left(z-d\right)}^{2}}{e}=1$$

**Figure 3:**
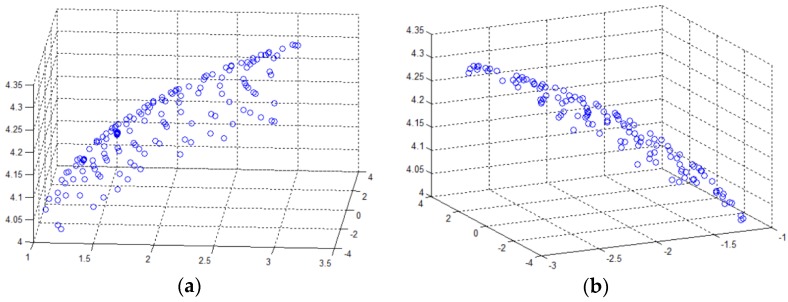
The coordinate distribution of voltage equivalent points at voltages of 0.71 V and −0.8 V: (**a**) at voltage of 0.71 V; (**b**) at voltage of −0.8 V*.*

The coefficients *a*, *b*, *c*, *d* and *e* are all related to the output voltage of the Hall sensor *V_Hall_*. Figure 3 shows the coordinate distribution at voltages of 0.71 V and −0.8 V, which shows two ellipsoid surfaces, respectively.

**Figure 4:**
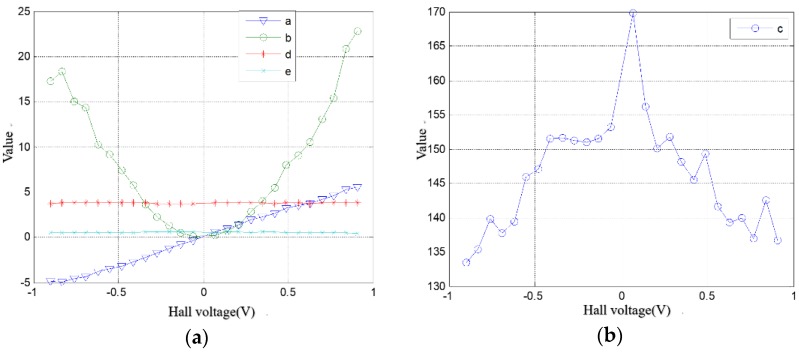
The curves of each ellipsoid equation parameter taking Hall sensor output voltage *V_Hall_* as an independent variable: (**a**) the curves of *a*, *b*, *d*, *e*; (**b**) The curve of *c.*

Then we used experimental data to fit the magnetic field by the ellipsoid function, at intervals of 0.07 V. Table 1 shows the ellipsoid equation parameters and mean square errors. Each parameter in the ellipsoidal equation is plotted with *V_Hall_* as an independent variable, as shown in Figure 4.

We observed the shape of the curves and selected the suitable curve to fit, and then the function expressions of each parameter were deduced:

(9) $$\left\{ \begin{array}{l} a=6.195V_{Hall}\\ b=26.910V_{Hall}{}^{2}+0.460\\ c=-22.840V_{Hall}{}^{2}+152.300\\ d=3.790\\ e=0.533 \end{array}\right.$$

Substituting Equation (9) into the ellipsoid model:

(10) $$\frac{{\left(x-6.195V_{Hall}\right)}^{2}}{26.910V_{Hall}{}^{2}+0.460}+\frac{y^{2}}{-22.840V_{Hall}{}^{2}+152.300}+\frac{{\left(z-3.790\right)}^{2}}{0.533}=1$$

In the proposed short stroke stage, six Hall sensors and six pairs of permanent magnets were utilized. Therefore, (10) can be rewritten as:

(11) $$\left\{ \begin{array}{l} x_{i}=f_{1i}\left(X_{m},Y_{m},Z_{m},\theta_{x},\theta_{y},\theta_{z}\right)\\ y_{i}=f_{2i}\left(X_{m},Y_{m},Z_{m},\theta_{x},\theta_{y},\theta_{z}\right)\\ z_{i}=f_{3i}\left(X_{m},Y_{m},Z_{m},\theta_{x},\theta_{y},\theta_{z}\right) \end{array}\right.\;i=1,2,\cdots ,6$$

Substituting Equation (11) into Equation (10) yields:

(12) $$\frac{{\left(x_{i}-6.195V_{Hall}{}_{i}\right)}^{2}}{26.910{V_{Halli}}^{2}+0.460}+\frac{y_{i}{}^{2}}{-22.840{V_{Halli}}^{2}+152.300}+\frac{{\left(z_{i}-3.790\right)}^{2}}{0.533}=1\;i=1,2,\cdots ,6$$

And the relationship between the voltage and the six DOF of the chuck can be obtained by applying two 6 × 6 matrices, *C* and *D*:

(13) $$V_{Hall}=CX_{G}+D$$

where

$$V_{Hall}={\left[V_{Hall1},V_{Hall2},V_{Hall3},V_{Hall4},V_{Hall5},V_{Hall6}\right]}^{T}\;X_{G}={\left[X_{m},Y_{m},Z_{m},\theta_{x},\theta_{y},\theta_{z}\right]}^{T}$$

With the aid of ellipsoid function, the relationship between the output voltages of the Hall sensors and the six-DOF displacements of the chuck was deduced, where the parameter matrixes were unknown. Therefore what we should focus on next is to solve the parameter matrixes.

## 4. Matrixes Solution

**Figure 5:**
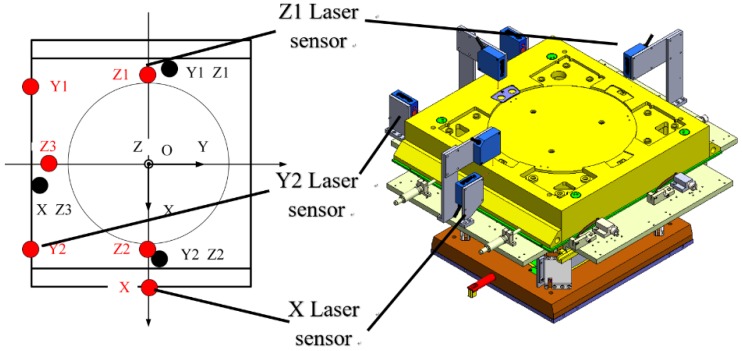
Schematic diagram of the calibration system and physical diagram of the calibration fixture consisting of laser sensors.

In order to obtain the relationship between the output voltage of the Hall sensors and the six-DOF displacements of the chuck, the matrixes *M* and *N* are required to be experimentally measured. We therefore used the laser displacement sensors to build an additional set of six DOF sensor systems to independently calibrate the Hall sensors. Figure 5 shows the schematic diagram of the calibration system and the corresponding positions of the laser sensors in the right physical diagram.

The displacement matrix of the center of the wafer is *X_G_*, the Hall sensor displacement matrix is *X_Hall_*, the Hall sensor output voltage matrix is *V_Hall_*, and the laser sensor displacement matrix is *X_Laser_*.

### 4.1. Known Conditions

1. *X_Laser_*, *V_Hall_* can be obtained through calibration fixtures. Assuming that the number of calibration data points is *N*, the known point sequences are then {*X_Laser_*_1_,⋯*X_Laserk_*,⋯*X_LaserN_*} and {*V_Hall_*_1_,⋯*V_Hallk_*,⋯*V_HallN_*}.
2. According to the mechanical structure of the device, the corresponding initial coordinates of Hall sensor probes are as follows (unit: mm):
  (14) $$\left\{ \begin{array}{l} X1(-53.35,\;191.35,-72.72)=(x_{h1},x_{h2},x_{h3})\\ Y1(-184.65,-10.65,-72.72)=(y_{h11},y_{h12},y_{h13})\\ Y2(184.65，-17.85，-72.72)=(y_{h21},y_{h22},y_{h23})\\ Z1(-181.3，\;14.0，-37.95)=(z_{h11},z_{h12},z_{h13})\\ Z2(181.30,\;14.50,-37.95)=(z_{h21},z_{h22},z_{h23})\\ Z3(-50.00,\;194.70,-37.95)=(z_{h31},z_{h32},z_{h33}) \end{array}\right.$$

The corresponding initial coordinates of Laser sensor probes are as follows (unit: mm):

(15) $$\left\{ \begin{array}{l} X(238,\;0,-11)=(x_{l1},x_{l2},x_{l3})\\ Y1(-159.8,-266,-11)\;=(y_{l11},y_{l12},y_{l13})\\ Y2(159.8,-266,-11\;)\;=(\;y_{l21},y_{l22},y_{l23})\\ Z1(-203,\;0,\;0)\;=(z_{l11},z_{l12},z_{l13})\\ Z2(203,\;0,\;0)=(\;z_{l21},z_{l22},z_{l23})\\ Z3(0,-190,\;0)\;=(\;z_{l31},z_{l32},z_{l33}) \end{array}\right.$$

### 4.2. Calibration Process

Make *X_GH_* and *X_GL_*, the displacements of wafer respectively obtained from the Hall sensors and laser sensors, as closest as possible. Analogous to the one-dimensional calibration work, make the vector sequences {*X_Laserk_*} and {*V_Hallk_*} as closest as possible:

(16) $$\min\left\{\sum_{k=1}^{N}{\left\|X_{GLk}-X_{GHk}\right\|}_{2}^{2}\right\}=\min\left\{\sum_{k=1}^{N}\sum_{i=1}^{6}{\left(x_{GLki}-x_{GHki}\right)}^{2}\right\}$$

The second-order norm of vector was used to compare the vector size. Obviously, when *X_GH_* and *X_GL_* are equal, the value of the above equation arrives at its minima. According to Equation (17), the output displacement *X_Laser_* of the laser sensors was used to solve the center of mass displacement *X_GL_*:

(17) $$X_{L\mathrm{aser}}=M_{L}X_{L}+X_{GL}$$

where

$$X_{Laser}={\left[x_{h},y_{h1},y_{h2},z_{h1},z_{h2},z_{h3}\right]}^{T},\;X_{GL}={\left[x_{g},y_{g},y_{g},z_{g},z_{g},z_{g}\right]}^{T},$$

$$X_{L}={\left[x_{l1},x_{l2},x_{l3},y_{l11},y_{l12},y_{l13},y_{l21},y_{l22},y_{l23},z_{l11},z_{l12},z_{l13},z_{l21},z_{l22},z_{l23},z_{l31},z_{l32},z_{l33}\right]}^{T},$$

$$M_{H}=[\begin{array}{cccccccccccccccccc} 1 & \theta_{z}+\theta_{x}\theta_{y} & \theta_{x}\theta_{z}-\theta_{y} & 0 & 0 & 0 & 0 & 0 & 0 & 0 & 0 & 0 & 0 & 0 & 0 & 0 & 0 & 0\\ & & & -\theta_{z} & 1-\theta_{x}\theta_{y}\theta_{z} & \theta_{x}+\theta_{y}\theta_{z} & & & & & & & & & & & & \\ & & & & & & -\theta_{z} & 1-\theta_{x}\theta_{y}\theta_{z} & \theta_{x}+\theta_{y}\theta_{z} & & & & & & & & & \\ & & & & & & & & & \theta_{y} & -\theta_{x} & 1 & & & & & & \\ & & & & & & & & & & & & \theta_{y} & -\theta_{x} & 1 & & & \\ & & & & & & & & & & & & & & & \theta_{y} & -\theta_{x} & 1 \end{array}]$$

From *X_GH_* = *X_GL_* (all indicated below by *X_G_*):

(18) $$X_{H\mathrm{all}}=M_{H}X_{H}+X_{GH}$$

where

$$X_{Hall}={\left[x_{h},y_{h1},y_{h2},z_{h1},z_{h2},z_{h3}\right]}^{T},\;X_{GH}=X_{GL}={\left[x_{g},y_{g},y_{g},z_{g},z_{g},z_{g}\right]}^{T},$$

$$X_{H}={\left[x_{h1},x_{h2},x_{h3},y_{h11},y_{h12},y_{h13},y_{h21},y_{h22},y_{h23},z_{h11},z_{h12},z_{h13},z_{h21},z_{h22},z_{h23},z_{h31},z_{h32},z_{h33}\right]}^{T}$$

$$M_{L}=[\begin{array}{cccccccccccccccccc} 1 & \theta_{z}+\theta_{x}\theta_{y} & \theta_{x}\theta_{z}-\theta_{y} & 0 & 0 & 0 & 0 & 0 & 0 & 0 & 0 & 0 & 0 & 0 & 0 & 0 & 0 & 0\\ & & & -\theta_{z} & 1-\theta_{x}\theta_{y}\theta_{z} & \theta_{x}+\theta_{y}\theta_{z} & & & & & & & & & & & & \\ & & & & & & -\theta_{z} & 1-\theta_{x}\theta_{y}\theta_{z} & \theta_{x}+\theta_{y}\theta_{z} & & & & & & & & & \\ & & & & & & & & & \theta_{y} & -\theta_{x} & 1 & & & & & & \\ & & & & & & & & & & & & \theta_{y} & -\theta_{x} & 1 & & & \\ & & & & & & & & & & & & & & & \theta_{y} & -\theta_{x} & 1 \end{array}]$$

Ignoring the rotation high-order harmonic components:

(19) $$X_{Laser}=T_{L}X_{G}+X_{L0}$$

where

$$T_{L}=\left[\begin{array}{cccccc} 1 & 0 & 0 & 0 & -x_{l3} & x_{l2}\\ 0 & 1 & 0 & y_{l13} & 0 & -y_{l11}\\ 0 & 1 & 0 & y_{l23} & 0 & -y_{l21}\\ 0 & 0 & 1 & -z_{l12} & z_{l11} & 0\\ 0 & 0 & 1 & -z_{l22} & z_{l21} & 0\\ 0 & 0 & 1 & -z_{l32} & z_{l31} & 0 \end{array}\right]\;X_{L0}=\left[\begin{array}{c} x_{l1}\\ y_{l12}\\ y_{l22}\\ z_{l13}\\ z_{l23}\\ z_{l33} \end{array}\right]$$

In a similar fashion, the displacement of the hall sensor can be expressed as follows:

(20) $$X_{Hall}=T_{H}X_{G}+X_{H0}$$

where

$$T_{H}=\left[\begin{array}{cccccc} 1 & 0 & 0 & 0 & -x_{h3} & x_{h2}\\ 0 & 1 & 0 & y_{h13} & 0 & -y_{h11}\\ 0 & 1 & 0 & y_{h23} & 0 & -y_{h21}\\ 0 & 0 & 1 & -z_{h12} & z_{h11} & 0\\ 0 & 0 & 1 & -z_{h22} & z_{h21} & 0\\ 0 & 0 & 1 & -z_{h32} & z_{h31} & 0 \end{array}\right]\;X_{H0}=\left[\begin{array}{c} x_{h1}\\ y_{h12}\\ y_{h22}\\ z_{h13}\\ z_{h23}\\ z_{h33} \end{array}\right]$$

After simplification:

(21) $$X_{Hall}=T_{H}T_{L}{}^{-1}\left(X_{Laser}-X_{L0}\right)+X_{H0}$$

By comparing Equation (13) with (21), we observe that *C* = *AT_H_* and *D* = *AX_H_*_0_ *+ B*, then Equation (13) can be expressed as:

(22) $$V_{Hall}=A\left(T_{H}X_{G}+X_{H0}\right)+B$$

(23) $$V_{Hall}=AX_{Hall}+B$$

After obtaining the displacement equation sequence {*X_Hallk_*} of the Hall sensor, the least squares method was used to find the parameter matrices *A* and *B*:

(24) $$\min\left\{\sum_{k=1}^{N}{\left\|V_{Hallk}-\left(AX_{Hallk}+B\right)\right\|}_{2}^{2}\right\}=\min\left\{P\right\}$$

where

$$V_{Hallk}={\left[v_{k1},v_{k2},v_{k3},v_{k4},v_{k5},v_{k6}\right]}^{T},\;X_{Hallk=}{\left[x_{k1},x_{k2},x_{k3},x_{k4},x_{k5},x_{k6}\right]}^{T}$$

and then substituting *A* and *B* expressions into (25):

(25) $$P=\sum_{k=1}^{N}{\left\|V_{Hallk}-\left(AX_{Hallk}+B\right)\right\|}_{2}^{2}=\sum_{k=1}^{N}\sum_{i=1}^{6}{\left(v_{ki}-\sum_{j=1}^{6}a_{ij}x_{kj}-b_{i}\right)}^{2}$$

According to the least squares method, make the partial derivative of the unknown be 0:

(26) $$\left\{ \begin{array}{l} \frac{\partial P}{\partial b_{q}}=-2\sum_{k=1}^{N}\left(v_{kq}-\sum_{j=1}^{6}a_{qj}x_{kj}-b_{q}\right)=0\\ \frac{\partial P}{\partial a_{pq}}={\left(\sum_{k=1}^{N}\sum_{i=1}^{6}{\left(v_{ki}-\sum_{j=1}^{6}a_{ij}x_{kj}-b_{i}\right)}^{2}\right)}^{\prime }=\sum_{k=1}^{N}2\left(v_{kp}-\sum_{j=1}^{6}a_{pj}x_{kj}-b_{p}\right)\left(-x_{kq}\right)=0 \end{array}\right.$$

In summary:

(27) $$\{\begin{array}{c} \sum_{k=1}^{N}v_{kq}-\sum_{k=1}^{N}\sum_{j=1}^{6}a_{qj}x_{kj}-Nb_{q}=0\left(q=1\cdots 6\right)\\ \sum_{k=1}^{N}x_{kq}v_{kp}-\sum_{k=1}^{N}\sum_{j=1}^{6}a_{pj}x_{kj}x_{kq}-\sum_{k=1}^{N}x_{kq}b_{p}=0\left({\left(p,q\right)}_{6\times 6}\right) \end{array}$$

Equation (27) is a system of linear equations consisting of 42 equations. The number of equations is equal to the unknown, so it can be solved. For simplicity of programing in the Matlab R2014a software, (27) it was rewritten as:

(28) $$\{\begin{array}{c} \sum_{j=1}^{6}\left(a_{qj}\cdot \sum_{k=1}^{N}x_{kj}\right)+Nb_{q}=\sum_{k=1}^{N}v_{kq}\left(q=1\cdots 6\right)\\ \sum_{j=1}^{6}\left(a_{pj}\cdot \sum_{k=1}^{N}x_{kj}x_{kq}\right)+b_{p}\cdot \sum_{k=1}^{N}x_{kq}=\sum_{k=1}^{N}x_{kq}v_{kp}\left({\left(p,q\right)}_{6\times 6}\right) \end{array}$$

The to-be-solved matrix of the equations is

(29) $$X=[a_{11},a_{12},a_{13}\ldots ,a_{65},a_{66},b_{1},b_{2},\ldots ,b_{6}]$$

The calibration is completed once the best parameters *A* and *B* are obtained. In this section, the relationship between the output voltage and the displacement of the Hall sensors is deduced, which provides a theoretical basis for decoupling the six-DOF data of the chuck.

## 5. Experimental Results and Analysis

**Figure 6:**
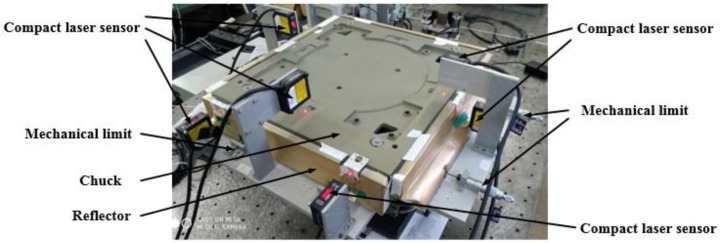
Experiment setups based on compact laser sensors.

Figure 6 is the experiment setup, which maintained mirrors around the chuck to measure the displacement of the laser sensors. The mechanical limits were used to adjust the chuck movement during calibrating.

**Figure 7:**
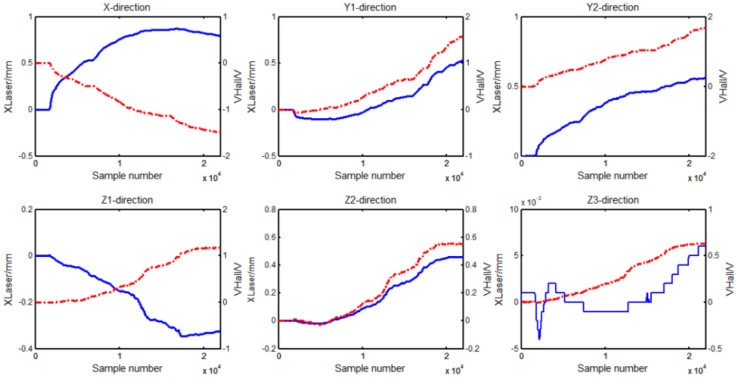
The laser sensors output displacement (blue) and the Hall sensors output voltage (red) sampled by the host computer.

**Figure 8:**
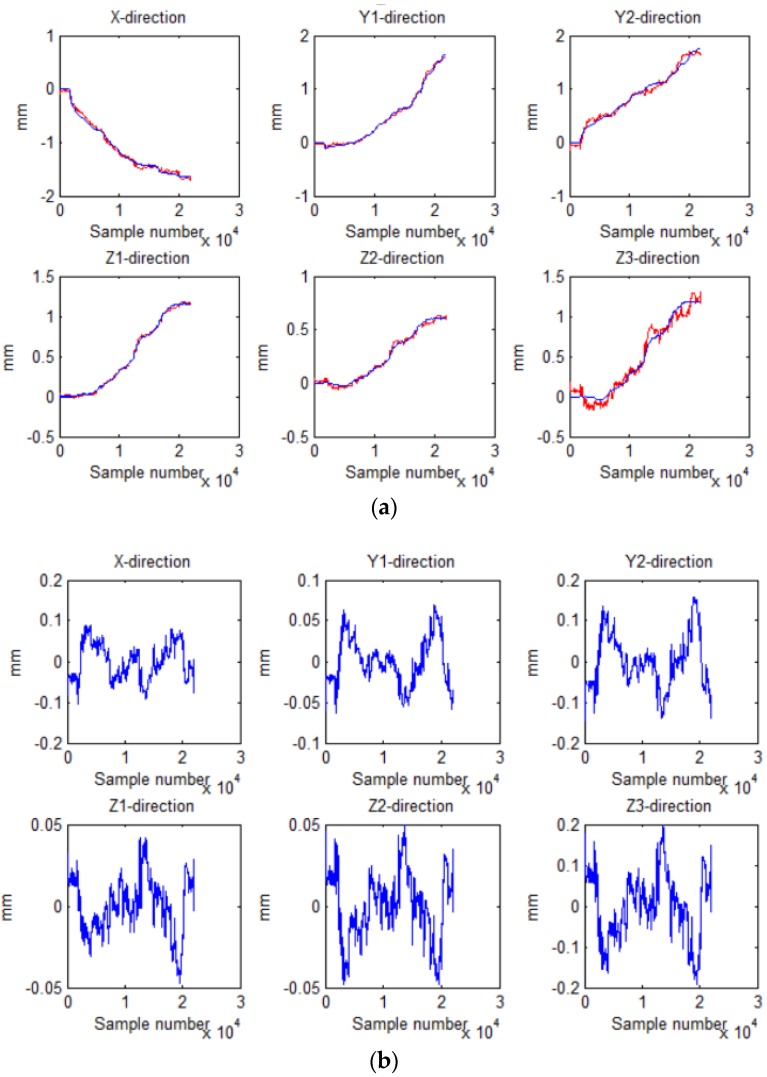
Displacement of the Hall sensors deduced by of the displacement of laser sensors and the least squares method: (**a**) displacement obtained by two ways; (**b**) difference between the two displacements.

Figure 7 shows the output displacement (blue) of laser sensors and the output voltage (red) of the Hall sensors sampled by the AD converter. Then the displacement of the Hall sensors (blue), deduced by the placement of laser sensors, was compared with the displacement (red) solved by least squares method, and the result is shown in Figure 8a. Figure 8b shows the errors between the two displacements. By comparison, it can be seen that the maximum error in the *X* direction was ±0.1 mm, in the *Y*1 and *Y*2 direction ±0.07 mm and ±0.15 mm, in both the *Z*1 and the *Z*2 direction ±0.05 mm, and in the *Z*3 direction ±0.2 mm.

**Figure 9:**
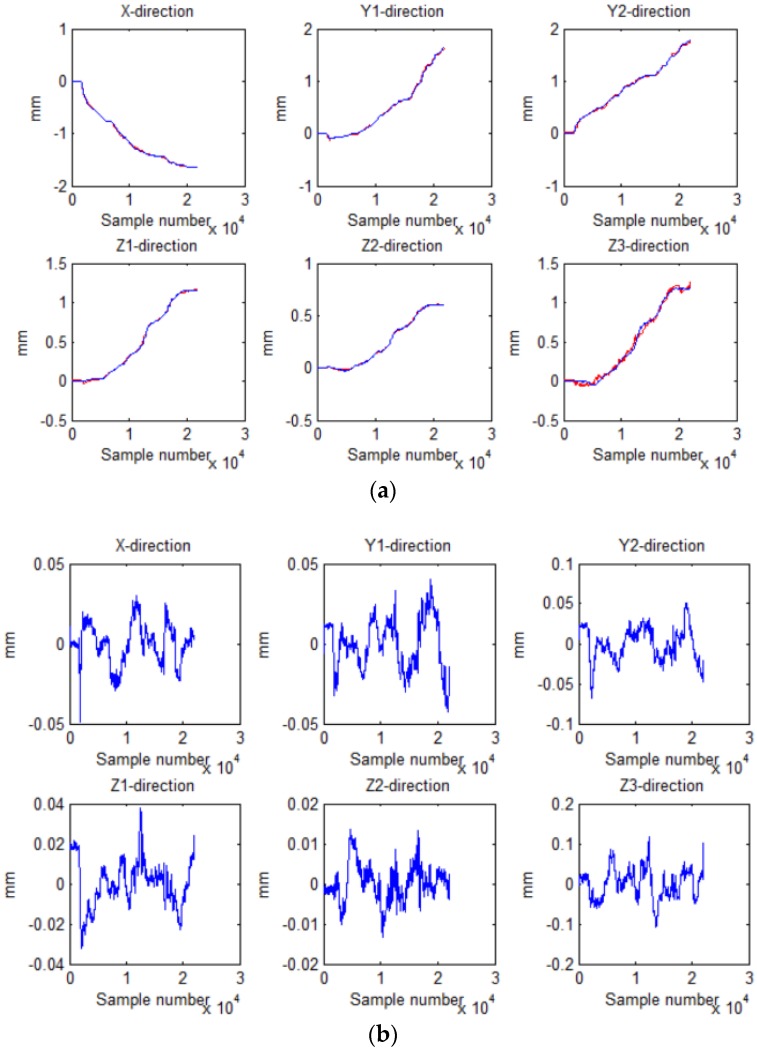
Displacement of the Hall sensors deduced by of the displacement of laser sensors and multidimensional fitting: (**a**) displacement obtained by two ways; (**b**) difference between the two displacements.

Further, the multidimensional fitting was performed, and the displacement of the Hall sensors (blue), deduced by the displacement of laser sensors displacement, was compared with the Hall displacement (red) solved by the least squares method, as shown in Figure 9a. And Figure 9b shows the errors. The maximum error in both the *X* and the *Y*1 direction was ±0.05 mm, in the *Y*2 direction ±0.06 mm, in the *Z*1 direction ±0.04 mm, in the *Z*2 direction ±0.02 mm, and in the *Z*3 direction ±0.12 mm. After comparison, it can be found that the error of the improved least squares method was much smaller than that of the previous method. The Hall displacement deduced by the laser sensor was closer to the displacement solved by the least squares method. Therefore, we choose multidimensional fitting to obtain parameters *A* and *B*.

The calibration results for parameters *A* and *B* are shown in Table 2 and Table 3.

**Figure 10:**
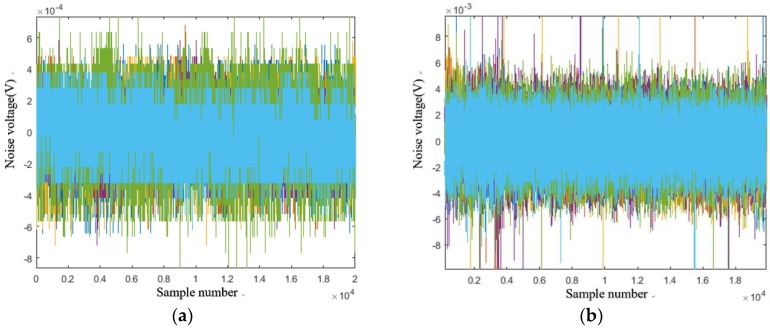
Inherent noise of the Hall sensor and the laser sensor: (**a**) inherent noise of the Hall sensor; (**b**) inherent noise of the laser sensor.

Figure 10 shows the inherent noise of the Hall sensor and the laser sensor, with the inherent noise peaks of 0.6 mV and 8 mV, respectively, so we ignored the inherent noise of the sensors in the experiment.

**Figure 11:**
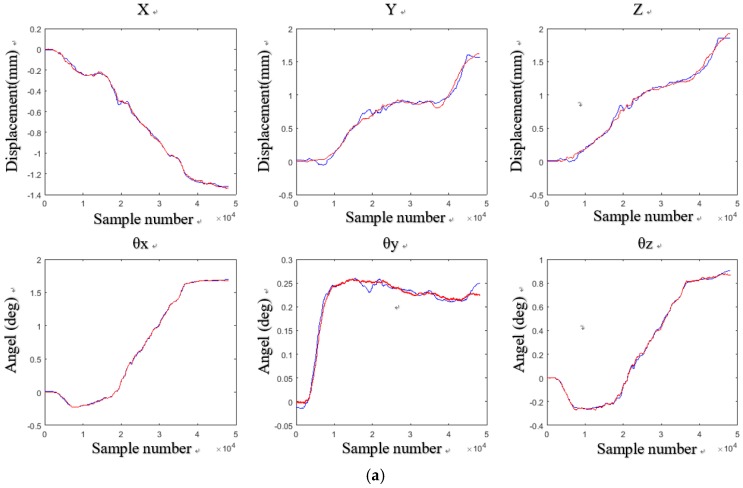

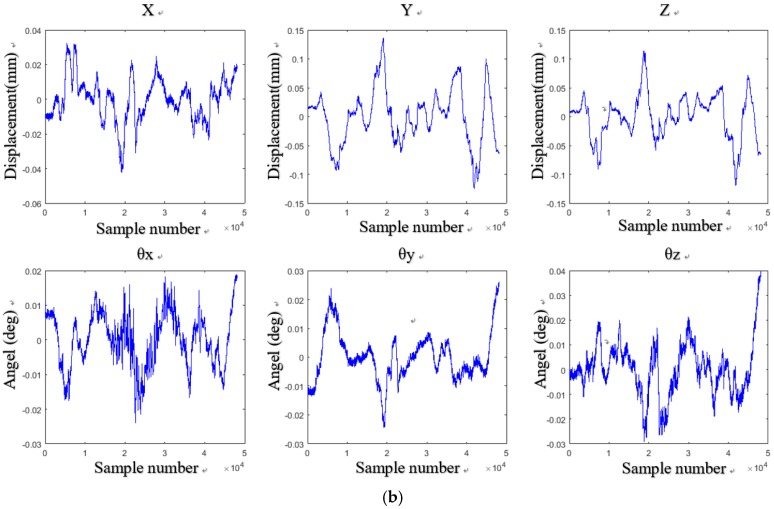
Six DOF measurement results and errors of the wafer stage at the first test: (**a**) six DOF measurement results; (**b**) six DOF measurement errors.

**Figure 12:**
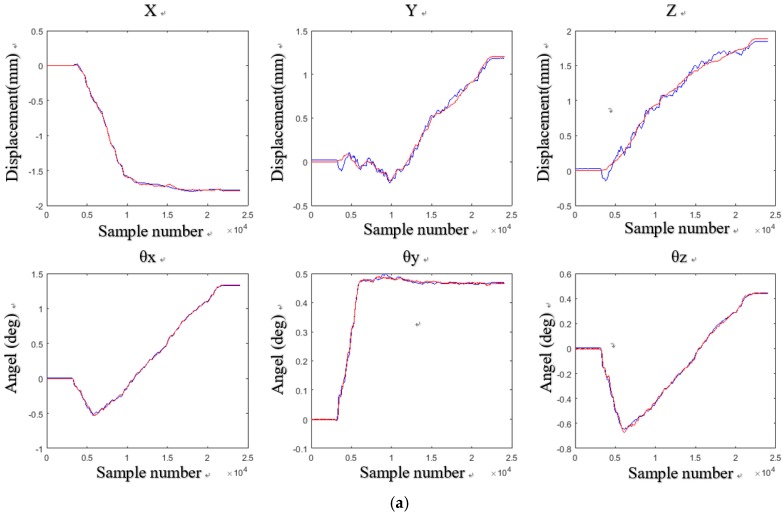

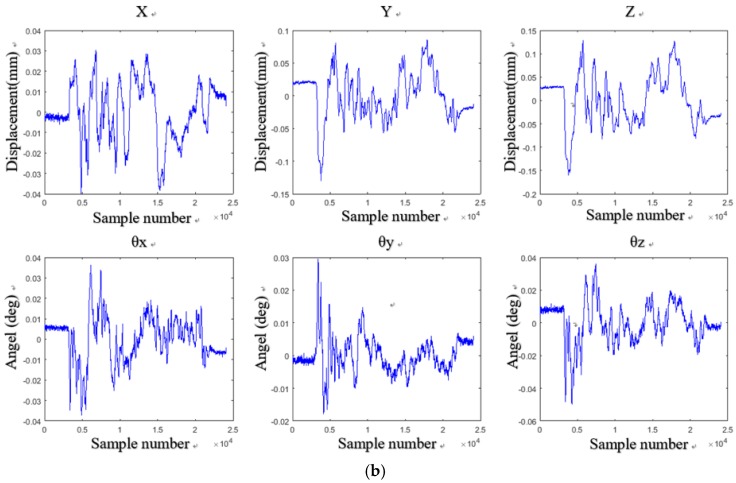
Six DOF measurement results and errors of the wafer stage at the second test: (**a**) six DOF measurement results; (**b**) six DOF measurement errors.

**Figure 13:**
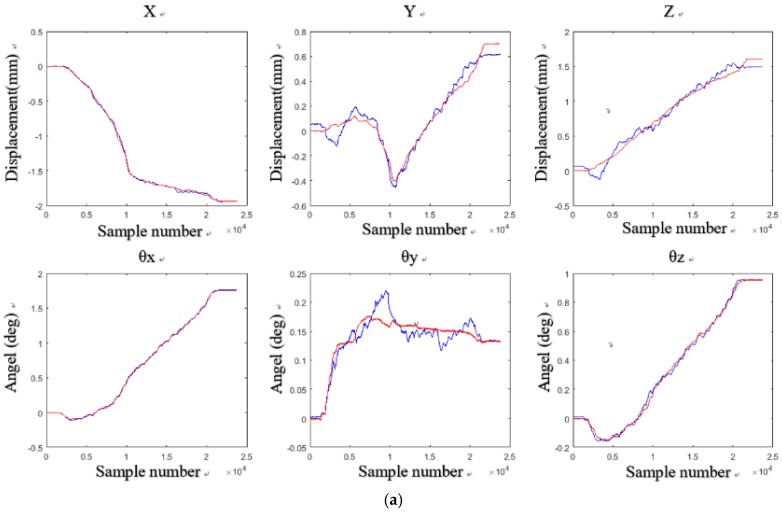

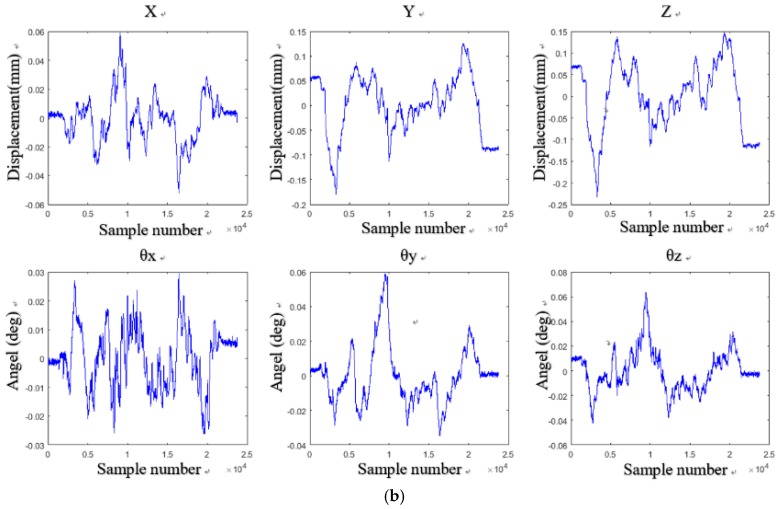
Six DOF measurement results and errors of the wafer Stage at the third test: (**a**) six DOF measurement results; (**b**) six DOF measurement errors.

After the calibration was completed, the wafer was tested again to obtain the six DOF displacement data. Through three tests on the experiment setups, we obtained the six DOF measurement results with the aid of the laser sensors and the Hall sensors. The measurement results and errors are shown in Figure 11, Figure 12 and Figure 13 respectively.

In Figure 11a, there is a displacement of −1.4 mm in the *X* direction, a displacement of 1.7 mm in the *Y* direction, and a displacement of 2 mm in the *Z* direction, *θ_x_*, *θ_y_*, and *θ_z_* are 1.7°, 0.25°, and 1°, respectively. In Figure 12a, when there is a displacement of −1.8 mm in the *X* direction, a displacement of 1.2 mm in the *Y* direction, and a displacement of 2 mm in the *Z* direction, *θ_x_*, *θ_y_*, and *θ_z_* are 1.5°, 0.5°, and 0.5°, respectively. In Figure 13a, when there is a displacement of −2 mm in the *X* direction, a displacement of 0.8 mm in the *Y* direction, and a displacement of 1.7 mm in the *Z* direction, *θ_x_*, *θ_y_*, and *θ_z_* are 1.7°, 0.25°, and 1°, respectively.

According to the measurement results, the six DOF measurement peak errors under different displacements are shown in Table 4. There was a peak error of 0.06 mm in the *X* direction, a peak error of 0.15 mm in the Y direction, and a peak error of 0.23 mm in the *Z* direction. The peak error of *θ_x_*, *θ_y_*, and *θ_z_* are 0.04°, 0.06°, and 0.07°. By analyzing the error of each degree of freedom, it can be noticed that the *Z*-direction, *θ_y_* and *θ_z_* measurement errors increase as the wafer displacement changes, while other degrees of freedom hardly change.

To sum up, the measurement accuracies of the six degrees of freedom measurement system are as follows: the maximum errors in the *X*, *Y,* and *Z* direction are 0.06 mm, 0.15 mm, and 0.23 mm, and the maximum errors of *θ_x_*, *θ_y_*, and *θ_z_* are 0.04°, 0.06°, and 0.07°.

## 6. Conclusions

In this paper, we present a six DOF displacement measurement methodology accomplished by six Hall sensors. Initially, the coordinate transformation was mathematically derived, which represented the relationships between the main coordinate system of the system and each body coordinate system of the Hall sensors. We can subsequently decouple the calculation to obtain the six-DOF displacements based on the ellipsoid function and the least squares method, where some elements in the transformation matrix are simplified due to the arrangement of the Hall sensors and the small angle of the rotating movement of the chuck. Finally, the experimental results demonstrate that the maximum errors of displacements can reach 0.23 mm and the maximum errors of inclinations can reach 0.07°.

The proposed methodology in this paper maintains several advantages. The physical meanings of the formula parameters are clear and easy to understand. We decoupled the calculations of the six-DOF displacements with the aid of the ellipsoid function and the least squares method easily. Moreover, the maximum errors in the six-DOF displacements and inclinations calculated during the exercise phase was very small.

## Figures and Tables

**Table 1:** The ellipsoid equation parameters and mean square error of the coordinates of the equivalent voltage points.

*V_Hall_* (V)	*a*	*b*	*c*	*d*	*e*	MSE (×10^−5^)
−0.9000	−4.8730	17.2287	133.4082	3.7653	0.4869	1.5882
−0.8300	−4.9842	18.3401	135.3778	3.7967	0.4841	2.2378
−0.7600	−4.5271	14.9682	139.7482	3.8022	0.4760	1.9036
−0.6900	−4.4135	14.3637	137.7598	3.8248	0.4749	2.4322
−0.6200	−3.7553	10.2714	139.4580	3.8035	0.4903	1.5774
−0.5500	−3.5330	9.2171	145.8678	3.8191	0.4897	2.4767
−0.4800	−3.1521	7.3729	147.0197	3.8213	0.5007	2.8644
−0.4100	−2.7517	5.7210	151.4560	3.8091	0.5302	1.9099
−0.3400	−2.1845	3.6350	151.6554	3.7735	0.5727	1.5192
−0.2700	−1.7248	2.2788	151.2365	3.7611	0.5942	1.0114
−0.2000	−1.2779	1.2677	150.9600	3.7432	0.6263	0.6854
−0.1300	−0.7836	0.4641	151.4696	3.7438	0.5976	0.7116
−0.0600	−0.3627	0.1004	153.1795	3.7372	0.6069	0.5898
0.0700	0.5670	0.2498	169.8377	3.8551	0.5516	2.6307
0.1400	0.9881	0.7461	156.1893	3.8146	0.5524	2.3675
0.2100	1.3435	1.3703	150.0127	3.7724	0.5773	1.5292
0.2800	1.9259	2.8212	151.7349	3.8086	0.5489	2.2555
0.3500	2.2771	3.9673	148.0845	3.7739	0.5801	1.6254
0.4200	2.6714	5.4854	145.5625	3.7626	0.5844	0.9534
0.4900	3.2424	7.9646	149.3453	3.8018	0.5338	1.9244
0.5600	3.4764	9.0506	141.6614	3.7855	0.5306	1.6327
0.6300	3.7473	10.5033	139.3210	3.7654	0.5412	1.2954
0.7000	4.1856	13.1069	139.8891	3.7720	0.5298	1.4367
0.7700	4.5792	15.3600	136.9817	3.8022	0.4724	2.2432
0.8400	5.2717	20.8156	142.4728	3.7998	0.4976	1.8012
0.9100	5.6072	22.8467	136.7349	3.8317	0.4365	1.4231

**Table 2:** Calibration result of parameter *A*.

*A_ij_* (V/mm)	*A_j_*_1_	*A_j_*_2_	*A_j_*_3_	*A_j_*_4_	*A_j_*_5_	*A_j_*_6_
*A*_1*j*_	−1.5397	−0.1037	−0.3315	−1.4055	−1.3080	−6.24751
*A*_2*j*_	0.5659	2.2981	0	1.0508	0.5097	11.1114
*A*_3*j*_	0.5148	0	3.6293	1.8000	1.3307	14.0375
*A*_4*j*_	0.3224	0.9862	−0.7829	−2.5066	0	0
*A*_5*j*_	0.2864	0.022993	−0.5654	0	1.4780	0
*A*_6*j*_	6.9354	5.1245	−12.6979	0	0	14.7979

**Table 3:** Calibration result of parameter *B*.

*B_ij_* (mm)	*B_j_*_1_	*B_j_*_2_	*B_j_*_3_	*B_j_*_4_	*B_j_*_5_	*B_j_*_6_
*B*_1*j*_	20.3677	−36.1377	−43.1450	3.9887	4.4567	−38.7739

**Table 4:** Six-DOF measurement peak errors.

	*X* (mm)	*Y* (mm)	*Z* (mm)	*θ_x_*	*θ_y_*	*θ_z_*
(1)	0.04	0.14	0.12	0.025°	0.025°	0.04°
(2)	0.04	0.13	0.16	0.04°	0.03°	0.05°
(3)	0.06	0.15	0.23	0.03°	0.06°	0.07°
